# Leukocyte telomere length, lipid parameters and gestational diabetes risk: a case-control study in a Chinese population

**DOI:** 10.1038/s41598-019-44968-9

**Published:** 2019-06-11

**Authors:** Qiao Weng, Keyong Deng, Fang Wu, Ming Gan, Jie Li, Yimin Dai, Yue Jiang, Jiaping Chen, Juncheng Dai, Hongxia Ma, Zhibin Hu, Hongbing Shen, Jiangbo Du, Yali Hu, Guangfu Jin

**Affiliations:** 1 Drum Tower Clinical Medical College, Nanjing Medical University, Nanjing, 210008 China; 2 Nanjing Drum Tower Hospital, Affiliated Hospital of Nanjing University Medical School, Nanjing, 210008 China; 3 Department of Epidemiology and Biostatistics, School of Public Health, Nanjing Medical University, Nanjing, 211166 China; 4 State Key Laboratory of Reproductive Medicine, Nanjing Medical University, Nanjing, 211166 China; 5 Department of Obstetrics and Gynecology, Nanjing Drum Tower Hospital, Affiliated to Nanjing University Medical School, Nanjing, 210008 China

**Keywords:** Molecular biology, Gestational diabetes

## Abstract

Telomere length (TL) is linked to various age-related diseases, but little is known about telomeres in gestational diabetes mellitus (GDM). We surveyed 509 subjects (113 GDM patients and 396 frequency matched controls) in Nanjing Drum Tower Hospital, Jiangsu province of eastern China. Relative telomere length (RTL) of genomic DNA extracted from peripheral blood leukocytes was measured using quantitative polymerase chain reaction (qPCR). Odds ratios (OR) and 95% confidence interval (CI) of GDM risk were calculated across tertiles of RTL using logistic regression model. Lipid parameters during the third trimesters of gestation (after 32 weeks) were collected from medical records. The general linear correlation test was used to explore the associations of lipid parameters with RTL. Our results showed that the RTL in GDM patients were significantly shorter than controls (0.302 ± 0.112 vs. 0.336 ± 0.164, *P* = 0.046). However, the GDM risk was significantly increased in subjects with median RTL (adjusted OR [aOR]: 1.936, 95% CI: 1.086, 3.453, *P* = 0.025) and the shortest RTL (aOR: 1.795, 95% CI: 1.004, 3.207, *P* = 0.048), compared to subjects with longest RTL. We also demonstrated that the lipid ratios (TC/TG, LDL/TG, HDL/TG, LDL/TC, TC/LDL) were significantly associated with RTL among controls. Overall, the present study indicated that attrition of telomeres would increase GDM risk among pregnant women, and the altered lipid levels may play an important role in RTL related GDM risk and pathogenesis.

## Introduction

Gestational diabetes mellitus (GDM), defined as a disease of glucose intolerance that leads to hyper-glycaemia with the first recognition during pregnancy^1^, is the most common metabolic abnormality and is increasing in prevalence worldwide^2^. Women with GDM are confronted with a great threat of diabetes, cardiovascular disease or hypertension later in life^3^. In addition, GDM also causes both immediate and long-term adverse consequences in the offspring^4^. Although several risk factors such as advanced maternal age, obesity^5^, family history of diabetes and specific racial backgrounds^6^ have been proved to be associated with GDM but the actual causes are still under investigation. It is widely accepted that the main pathological mechanisms of GDM involve the biochemical pathways resulting in insulin resistance and low-grade inflammation, in which oxidative stress plays a key role^7,8^.

Telomere length, an established biomarker of oxidative stress, has been associated with various age-related disorders (e.g. malignant tumor^9^, and infections^10^). Therefore, it has been proposed as a systemic marker for the development and progression of those biological aging diseases^11^. There are mounting evidences supporting associations between short telomeres and diabetes^12,13^. Hyperglycemia in diabetes status is known to increase oxidative stress, and then accelerates the telomere length shortening^14^. However, the studies involved in association between telomere length and gestational diabetes mellitus (GDM) are scarce^15^.

Circulating lipid patterns in GDM have been extensively studied, dyslipidemia is widely reported to be associated with the risk of developing GDM^16^. But the results of dyslipidemia in diabetic pregnancy are various due to different trimesters. Recently, a cross-sectional study suggested that metabolic syndrome and its components (e.g. lipid profiles) have also been associated with short telomere length^17^. To our knowledge, no studies have been carried out on the associations between GDM risk, lipid parameters, and telomere length. Hence, we hypothesize that altered lipid patterns may impose an influence on telomere length related GDM risk modification.

The prevalence of GDM in China is also alarming. A recent review systematically suggested the high incidence rate of GDM among women in mainland China^18^. In short, given the inconclusive evidence, we design a case-control study with 113 gestational diabetes mellitus (GDM) cases and 396 controls in Chinese population to evaluate the association between telomere length and GDM risk, and further to examine whether lipid metabolism plays a role in the relations between telomere length and GDM development.

## Results

The baseline characteristics of 113 GDM cases and 396 controls were shown in Table 1. There were no differences in maternal age at delivery, BMI (body mass index, Kg/m^2^), secondhand smoking status, pregnant experience and physical activity, total cholesterol (TC), low density lipoprotein (LDL), high density lipoprotein (HDL), triglycerides (TG) and fasting blood glucose (FBG) between these two groups (*P* > 0.05). In contrast, the significant differences were observed both in 1-hour and 2-hour postprandial blood glucose among groups (*P* < 0.001). Participants within GDM cases exhibited shorter RTL compared to those in control cases (0.302 ± 0.112 vs. 0.336 ± 0.164, *P* = 0.046). Strikingly, in subgroups divided by different characteristics, we also detected a similar trend (Table 2).

**Table 1:** Baseline characteristics of study participants.

Variables	Cases	Controls	*P*
	N(%)	N(%)	
Overall	113	396	
**Maternal age at delivery (years)**			
<25	15 (13.27)	62 (15.66)	0.059^a^
25~30	51 (45.13)	212 (53.54)	
30~35	32 (28.32)	97 (24.49)	
≥35	15 (13.27)	25 (6.31)	
**BMI (kg/m** ^**2**^ **)**			
<25%	23 (20.35)	98 (24.75)	0.149^a^
25~50%	28 (24.78)	100 (25.25)	
50~75%	19 (19.47)	99 (25.00)	
≥75%	40 (35.40)	99 (25.00)	
**Maternal exposure to secondhand smoke**			
≥Half an hour a day	9 (7.96)	27 (6.82)	0.310^a^
<Half an hour a day	21 (18.59)	101 (25.51)	
Never	83 (73.45)	268 (67.67)	
**Pregnant experience**			
Ever	51 (45.13)	167 (42.17)	0.575^a^
Never	62 (54.87)	229 (57.83)	
**Regular exercises**			
Daily	6 (5.31)	17 (4.29)	0.318^a^
3~6 Times per week	3 (2.65)	27 (6.82)	
1~2 Times per week	28 (24.78)	109 (27.53)	
Never	76 (67.26)	243 (61.36)	
RTL (Mean ± SD)	0.302 ± 0.112	0.336 ± 0.164	0.046^b^
Gestation weeks of RTL (Mean ± SD)	37.562 ± 1.319	37.820 ± 1.890	0.225^b^
Gestation weeks of lipid (Mean ± SD)	32.482 ± 1.344	32.843 ± 3.077	0.284^b^
TC (Mean ± SD)	5.681 ± 1.084	5.927 ± 1.252	0.136^b^
TG (Mean ± SD)	2.754 ± 1.037	2.790 ± 1.417	0.841^b^
LDL (Mean ± SD)	2.757 ± 0.820	2.897 ± 0.919	0.252^b^
HDL (Mean ± SD)	1.666 ± 0.420	1.728 ± 0.426	0.284^b^
FBG (Mean ± SD)	4.568 ± 0.530	4.511 ± 0.361	0.232^b^
1-hour PBG (Mean ± SD)	9.881 ± 1.384	8.489 ± 1.204	<0.01^b^
2-hour PBG (Mean ± SD)	8.482 (1.295)	6.790 ± 0.833	<0.01^b^

Abbreviations: BMI, body mass index; RTL, relative telomere length; FBG, fasting blood glucose; PBG, postprandial blood glucose; SD, standard deviation; TC, total cholesterol; TG, triglycerides; LDL, low density lipoprotein cholesterol; HDL, high density lipoprotein cholesterol.

^a^*P* value for *χ2-test* that was used to compare the differences between variables.

^b^*P* value for paired *t-test* that was used to compare the differences between variables.

**Table 2:** Distributions of characteristics and RTL between GDM cases and controls.

Variables	Cases	RTL Mean ± SD	Controls	RTL Mean ± SD	*P* ^a^
	N (%)		N (%)		
Overall	113		396		
**Maternal age at delivery** (**years)**					
<25	15 (13.27)	0.360 ± 0.163	62 (15.66)	0.355 ± 0.208	0.940
25~30	51 (45.13)	0.227 ± 0.082	212 (53.54)	0.338 ± 0.165	0.014
30~35	32 (28.32)	0.297 ± 0.071	97 (24.49)	0.315 ± 0.132	0.482
≥35	15 (13.27)	0.341 ± 0.176	25 (6.31)	0.352 ± 0.137	0.829
**BMI (kg/m** ^**2**^ **)**					
<25%	23 (20.35)	0.326 ± 0.147	98 (24.75)	0.338 ± 0.161	0.759
25%~50%	28 (24.78)	0.290 ± 0.078	100 (25.25)	0.337 ± 0.213	0.089
50%~75%	22 (19.47)	0.321 ± 0.105	99 (25.00)	0.345 ± 0.156	0.512
≥75%	40 (35.40)	0.285 ± 0.114	99 (25.00)	0.324 ± 0.111	0.081
**Maternal exposure to secondhand smoke**					
≥Half an hour a day	9 (7.96)	0.283 ± 0.075	27 (6.28)	0.367 ± 0.210	0.257
<Half an hour a day	21 (18.59)	0.293 ± 0.099	101 (25.51)	0.361 ± 0.222	0.173
Never	83 (73.45)	0.307 ± 0.121	268 (67.67)	0.326 ± 0.132	0.289
**Pregnant experience**					
Ever	51 (45.13)	0.315 ± 0.129	167 (42.17)	0.335 ± 0.152	0.422
Never	62 (54.87)	0.291 ± 0.095	229 (57.83)	0.337 ± 0.172	0.054
**Regular exercises**					
Daily	6 (5.31)	0.307 ± 0.093	17 (4.29)	0.258 ± 0.138	0.431
3~6 Times per week	3 (2.65)	0.238 ± 0.047	27 (6.82)	0.307 ± 0.127	0.366
1~2 Times per week	28 (24.78)	0.312 ± 0.091	109 (27.53)	0.307 ± 0.124	0.874
Never	76 (67.26)	0.300 ± 0.123	243 (61.36)	0.358 ± 0.181	0.013

^a^Derived from t-test for RTL between cases and controls.

Logistic regression was used to estimate adjusted OR (aOR) and 95% confidence interval(95% CI) for GDM risk with RTL. When compared with subjects in the highest (longest) tertile group, significant elevated risk of GDM was observed in the subjects from the median tertile (aOR: 1.936, 95% confidence interval [95% CI]: 1.086–3.453, *P* = 0.025) and the lowest (shortest) tertile (aOR: 1.795, 95% CI: 1.004–3.207, *P* = 0.048) groups, with adjustment for maternal age at delivery (Table 3).

**Table 3:** Association between RTL and GDM risk among Chinese women.

Grouping of RTL by controls category	Cases, N (%)	Controls, N (%)	OR (95% CI)	*P*	OR (95% CI)^a^	*P* ^a^	
Tertile	≥0.355	23 (21.9)	121 (33.3)	1.000 (reference)	—	1.000 (reference)	—
	0.264~0.355	42 (40.0)	121 (33.3)	1.826 (1.035, 3.221)	0.038	1.936 (1.086, 3.453)	0.025
	<0.264	40 (38.1)	121 (33.3)	1.739 (0.982, 3.080)	0.058	1.795 (1.004, 3.207)	0.048

^a^Derived from logistic regression with an adjustment for Maternal age at delivery(years).

Given the observed reverse correlation between GDM risk and RTL, further retrospective analysis was conducted to investigate the associations between altered lipid parameters in the third trimesters of gestation and RTL. As a result, no significant correlations were observed (Supplementary Table 1). Considered about the changes in lipid ratios have been shown as a better indicator of disease risk than changes in absolute levels of lipids or lipoproteins, we examined the general linear correlations between lipid ratios and RTL. We observed significant positive correlations between TC/TG ratio (*r* = 0.215, *P* = 0.002), LDL/TG ratio (*r* = 0.283, *P* < 0.001), HDL/TG ratio (*r* = 0.162, *P* = 0.021), LDL/TC ratio (*r* = 0.214, *P* = 0.002), but an inverse correlation was found between TC/LDL ratio (*r* = −0.185, *P* = 0.008) (Table 4). The correlations between lipid ratios and RTL were also shown in (Supplementary Fig. 1).

**Table 4:** Correlation between lipid parameter ratios† and RTL among controls.

	TC	TG	LDL	HDL
TC	—	r = 0.215; P = 0.002	r = −0.185; P = 0.008	r < 0.001; P = 0.998
TG	r = −0.101; P = 0.150	—	r = 0.121; P = 0.086	r = −0.077; P = 0.276
LDL	r = 0.214; P = 0.002	r = 0.283; P < 0.001	—	r = 0.080; P = 0.256
HDL	r = −0.025; P = 0.722	r = 0.162; P = 0.021	r = 0.120; P = 0.089	—

^†^Lipid ratios are calculated by lipid parameters at the header column (the left of the table) divided by lipid parameters at the header row (the upper of the table) of the table.

## Discussion

The present case-control analysis was conducted to evaluate the associations of GDM with telomere length from peripheral blood leukocytes and lipid profiles on the ground of Chinese women population. We found that participants within GDM cases showed shorter RTL compared to those in control cases, which also suggested that shorter telomere length was related to the increase of GDM risk. In view of the effects of lipid on RTL, we also observed significantly correlations between TC/TG, LDL/TG, HDL/TG, LDL/TC ratios and RTL.

A previous study was conducted with 25 cases of GDM and 50 controls in Washington State. *Harville et al*.^15^ showed a trend of shorter telomeres in women with GDM, although the association was not statistically significant, which may be explained by the limited sample size, or the variability of leukocyte between individuals. Several previous studies also reported that reduced leukocyte telomere length was associated with incident diabetes risk^12,13^. In addition, shorten telomere length in GDM subjects has been shown in cord blood^19^ or in placentas^20^. While inflammation and oxidative stress have been postulated as crucial contributors to diabetes including GDM, telomere attrition can aggravate the chronic inflammation^21^ or oxidative stress^22^ of individuals. Higher plasma glucose status, which in turn can increase oxidative stress to accelerate the telomere length shortening and good glycemic control was associated with more favorable telomere dynamics^23^. Therefore, the relationship between decreased telomere length and increased GDM risk in our current study is biologically eligible. Meanwhile, since the blood samples were collected within 1 week before delivery, the possibility of GDM-induced telomere length shortening should not be excluded. Therefore, a well-designed study is necessary for further confirming the association between GDM risk and telomere length.

As a biomarker of aging, telomeres are also recognized to be influenced by the subject’s chronological age^11,24^. We observed a trend of negative relation between RTL and maternal age at delivery, but the result was not significant, which may be due to the narrow range of maternal age in our study. The most persuasive evidence indicated that impacts on telomere attrition with aging is relatively rapid during childhood and adolescence, while stable in adulthood^25,26^. In addition, pregnant women may be more likely to keep a healthy lifestyle and escape from adverse exposures. An experimental study with 5-year follow-up has suggested that comprehensive lifestyle intervention may promote the lengthening of telomere length^27^. The above interpretations may underestimate the adverse effects of aging on telomere length in our study.

Recently, metabolic syndrome was confirmed as a precursor to GDM, with abnormal lipid metabolism being associated with high risk of GDM^16,28^. In the present study, the comparisons of baseline TC, TG, LDL, HDL between cases and controls were not significant. Existing study also showed that there are no significant differences in any of lipid profiles between GDM and controls^29^. However, in diabetic or normal pregnancy, the results of lipid patterns are not universal. For instance, the cholesterol level in the third trimester of gestation were lower, unchanged or even higher in different studies^29,30^. These discrepant results may be owing to some confounders such as lifestyle habits, ethnicity, different collection protocols and genetic risks for GDM, as those factors are important sources of heterogeneity in the relationship between lipid levels and GDM. In the present study, all the lipid profiles were retrospectively collected following the criteria: the third trimesters (after 32 weeks) based on the medical records, regularly visiting the clinic, and healthier lifestyle, which could exclude the effects of lipid profiles on GDM. Lipid metabolism could be stable while telomere length manifested shortening in GDM cases, which was in accordance with the results from type 2 diabetes^31^, indicating that telomere length shortening may be sensitive and potentially could be a biomarker in the early stage of GDM development.

To the best of our knowledge, lipid ratios have been shown as better indicators for risk assessment and progression of certain disease than individual lipid profiles themselves^32,33^. Herein, we hypothesized that altered lipid ratios could indicate the alteration of telomere length. In the present study, there are positive correlations between RTL and TC/TG, LDL/TG, HDL/TG, LDL/TC ratios, an inverse correlation between RTL and TC/LDL ratio. Lipid ratios were more comprehensive than assessing the association between single lipid parameter and RTL, which may avoid the fluctuation of single parameter among individuals, and reflect the disease or health status more objectively. Previous studies demonstrated that HDL exerts anti-oxidant and anti-inflammatory effects and TG reflects the accruing burden of oxidative stress and inflammation^17^. They found that higher TG and lower HDL were associated with shorter telomere length. Furthermore, the elevated TC levels may cause augmented cell turnover and increased production of reactive oxygen species (ROS)^34^ and increased LDL levels were closely associated with decreased serum total antioxidant status (TAS)^35,36^. Thus, our results of correlations between lipid ratios and RTL were partially in consistency with these previous studies. Nevertheless, we firstly reported that correlations between RTL and each lipid ratio were weak in GDM cases. Further studies should focus on validating the mechanism of lipid parameters on telomeres in GDM.

This study has important strengths and limitations. The advantage is that we firstly observed the contribution of telomere length to the risk of GDM among Chinese women and revealed that shorten telomere length was significantly related to higher GDM risk, and ratios for lipid parameter may be better for evaluating on its association with telomere length than single lipid parameter. Nonetheless, there were still some potential limitations required discussion. Firstly, lipid parameters in this study were obtained during late pregnancy while blood samples for telomere length measuring were collected before childbirth, this unsynchronized data may cause potential bias on our analysis. Secondly, as any of case-control study, we could not confirm the exact causation between shorter telomere length and GDM. Thirdly, since we tested samples during the last trimester of gestation, the effect of GDM on telomere length and lipid profiles was hard to evaluate. Moreover, there is substantial evidence that it is the shortest telomeres that leads to telomere dysfunction. However, although the qPCR method is widely used in population study, which can only provide a mean relative telomere length. Further studies with more accurate telomere length measuring method may provide more reliable evidences^37^.

In conclusion, our study provided some valuable clues for further exploration on GDM risk and pathogenesis.

## Methods

### Study recruitment

GDM cases and controls were recruited from Nanjing Drum Tower Hospital in Nanjing, Jiangsu province of eastern China between April 2014 and April 2015. According to the guidelines from the International Association of Diabetes and Pregnancy Study Groups (IADPSG), GDM is diagnosed by specialist doctors^38^. During the same period, the women who also presented for routine GDM screening but in normoglycemic status were selected as controls. As a result, 113 cases and 396 age frequency matched controls were included in current study. After writing informed consent, each participant was interviewed by a well-trained interviewer with a structured questionnaire. Data available in the questionnaire include demographic data and lifestyle related factors such as cigarette exposure, physical activity, alcohol consumption, tea or coffee drinking, medical and reproductive characteristics etc. Full details of lipid parameters in the last trimesters of gestation were retrospectively extracted from medical records, including total cholesterol (TC), triglycerides (TG), low density lipoprotein (LDL), high density lipoprotein (HDL), and fasting blood glucose (FBG). Overall, 509 pregnant women provided venous blood samples within 1 week before delivery. Blood samples were shipped by cold chain equipment to the study laboratories and stored until analysis. Ethical approval for the study was obtained from the ethics committees of Nanjing Medical University and Nanjing Drum Tower Hospital and this study complied with the Declaration of Helsinki.

### Relative telomere length measurement

Genomic DNA was extracted from leukocytes of peripheral blood. Telomere length was measured on a modified quantitative polymerase chain reaction (qPCR) by ABI PRISM 7900HT Sequence Detection System (Applied Biosystems), which was previously described in our study^39^. This method expressed telomere length as a ratio of telomere repeat length (T) to single-gene copy number (S)(T/S). A lower T/S ratio reflects shorter RTL. The reference DNA (pooled from 5 healthy controls) was used to generate a standard curve for quantification. After exclusion of outliers, average cycle threshold (Ct) values of the remaining samples were calculated. Telomere PCR systems included primer TEL1,5′-GGTTTTTGA[GGGTGA]4GGGT-3′ and primer TEL2,5′-TCCCGACTAT[CCCTAT]4CCCTA-3′. The single-copy gene(36B4) primers included 36B4u, 5′-CAGCAAGTGGGAAGGTGTAATCC-3′, and 36B4d, 5′-CCCATTCTATCATCAACGGGTACAA-3′. Samples were performed in duplicate in 384-well plate and mean was used for calculations. Each reaction system contains 10 μl SYBR ® Green PCR Master Mix (Applied Biosystems) and a 5 ng/µl of DNA. Personnel of the laboratory that measured LTL were blinded to characteristics of the participants. RTL was calculated on the basis of Cawthon’s formula^40,41^:

$${2}^{-({\rm{\Delta }}\mathrm{Ct}1-{\rm{\Delta }}\mathrm{Ct}2)}={2}^{-{\rm{\Delta }}{\rm{\Delta }}\mathrm{Ct}}$$

### Statistical analysis

Analyses were all performed with Stata version 9.2 (Stata Corp, College Station, TX). Sample characteristics were described as Means and SDs, or percentages. Pairwise comparisons for categorical variables (maternal age at delivery, secondhand smoking status, physical activity) were performed using Pearson Chi-square test. Continuous variables were analyzed by Paired t-test. To assess the associations of RTL on the risk of GDM, Logistic regression model was used to estimate odds ratios (ORs) and 95% confidence interval (CI) for GDM risk with RTL (categorized as RTL of <0.264, 0.264–0.355, ≥0.355), adjusting for maternal age at delivery. In addition, Linear correlation test was used to test the association of lipid profiles with RTL among controls. All statistical tests were two-sided. *P* value < 0.05 considered significant.

## Supplementary information

Leukocyte telomere length, lipid parameters and gestational diabetes risk: a case-control study in a Chinese population.

## Data Availability

The datasets employed to support this study are available from the corresponding author on reasonable request.
