# Supplementary material for: Leukocyte telomere length, lipid parameters and gestational diabetes risk: a case-control study in a Chinese population
Source: Sci Rep. 2019 Jun 11;9:8483. doi: 10.1038/s41598-019-44968-9 (PMC6560111; doi:10.1038/s41598-019-44968-9)
Supplement: Supplementary file 1 — Leukocyte telomere length, lipid parameters and gestational diabetes risk: a case-control study in a Chinese population. [file 41598_2019_44968_MOESM1_ESM.pdf]

Leukocyte telomere length, lipid parameters and gestational diabetes risk: a case-control study in a Chinese population.

Qiao Weng [1,2,5†], Keyong Deng [3†], Fang Wu [3†], Ming Gan[3], Jie Li [1,5], Yimin Dai[1,2,5], Yue Jiang [3], Jiaping Chen [3], Juncheng Dai [3], Hongxia Ma [3], Zhibin Hu [3,4], Hongbing Shen [3,4], Jiangbo Du [3,4*], Yali Hu [1,2,5*], Guangfu Jin [3,4*]

Affiliations:

[1] Drum Tower Clinical Medical College, Nanjing Medical University, Nanjing 210008, China

[2] Nanjing Drum Tower Hospital, Affiliated Hospital of Nanjing University Medical School, Nanjing 210008, China

[3] Department of Epidemiology and Biostatistics, School of Public Health, Nanjing Medical University, Nanjing 211166, China

[4] State Key Laboratory of Reproductive Medicine, Nanjing Medical University, Nanjing 211166, China

[5] Department of Obstetrics and Gynecology, Nanjing Drum Tower Hospital, Affiliated to Nanjing University Medical School, Nanjing 210008, China

Supplementary Table 1. Correlations between RTL and other variables among controls.

Variables	*r*	*P*
Maternal age	-0.029	0.579
TC	-0.017	0.815
TG	-0.040	0.568
LDL	0.064	0.365
HDL	-0.056	0.432
FBG	-0.019	0.774
1-hour PBG	-0.095	0.150
2-hour PBG	-0.019	0.768

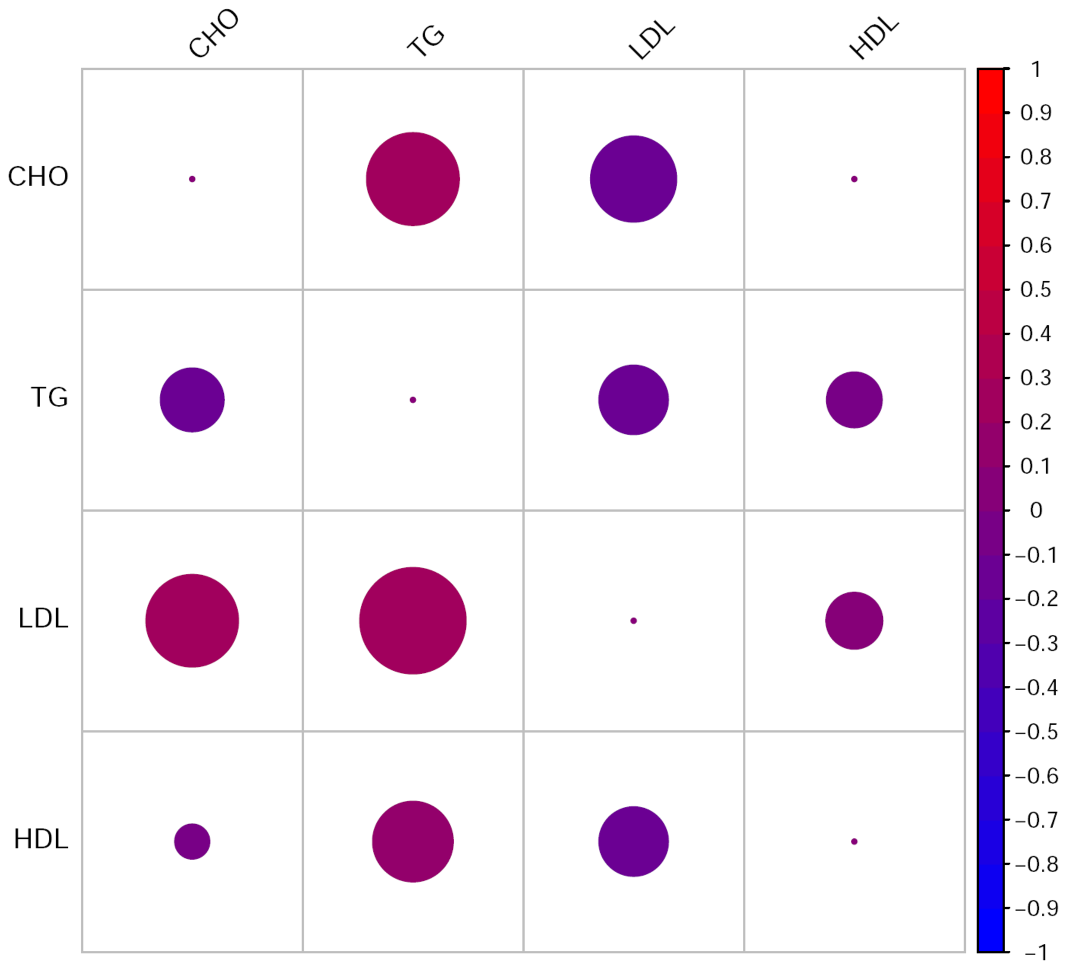

Supplementary Figure1. Correlations between lipid ratios and RTL. The scale to the right of the figure indicates the strength of the correlation, with darker and larger dots indicating stronger correlation (Red: Positive correlation; Blue: Negative correlation). Lipid ratios are calculated by lipid profiles (the left of the figure) to the others (the upper of the figure).
